# How to assess communication skills? Development of the rating scale ComOn Check: Evaluation of communication skills

**DOI:** 10.1080/10872981.2017.1392823

**Published:** 2017-11-15

**Authors:** K. Radziej, J. Loechner, C. Engerer, M. Niglio de Figueiredo, J. Freund, H. Sattel, C. Bachmann, P. O. Berberat, A. Dinkel, A. Wuensch

**Affiliations:** a Psychosomatic Medicine and Psychotherapy, Klinikum rechts der Isar, Technical University of Munich, Munich, Germany; b Department of Child and Adolescent Psychiatry, Psychotherapy, and Psychosomatics, Ludwig-Maximilians-Universität, Munich, Germany; c TUM Medical Education Center, TUM School of Medicine, Klinikum rechts der Isar, Technical University of Munich, Munich, Germany; d Department of General, Visceral, and Transplantation Surgery, University Hospital Heidelberg, Heidelberg, Germany; e Center for Mental Health, Department of Psychosomatic Medicine and Psychotherapy, Medical Center, University of Freiburg, Faculty of Medicine, University of Freiburg, Freiburg, Germany; f Clinic of Dermatology and Venereology, Medical Center, University of Freiburg, Faculty of Medicine, University of Freiburg, Freiburg, Germany; g Institute of Medical Education, Faculty of Medicine, University of Bern, Bern, Switzerland

**Keywords:** Communication skills training, rating scale, assessment tool, medical education, OSCE

## Abstract

**Background**: Good communication is a core competency for all physicians. Thus, medical students require adequate preparation in communication skills. For research purposes, as well as for evaluation in teaching, there is a clear need for reliable assessment tools. We analyzed the shortcomings of existing instruments and saw a need for a new rating scale. The aim of this publication is to describe the development process for, and evaluation of, a new rating scale.

**Methods**: First, we developed the rating scale in 10 steps. Then, two raters evaluated the newly developed rating scale by rating 135 videotaped consultations of medical students with standardized patients. Additionally, standardized patients evaluated students’ performance, which was used as an outside criterion to validate ratings.

**Results**: Our rating scale comprises six domains with 13 specific items evaluated on a five-point Likert scale: initiating conversation, patient’s perception, structure of conversation, patient’s emotions, end of conversation, and general communication skills. Item-total correlation coefficients between the checklist items ranged from 0.15 to 0.78. Subscale consistency was calculated for domains comprised of more than one item and Cronbach’s *α* ≥ 0.77, indicating acceptable consistency. Standardized patients’ global evaluation correlated moderately with overall expert ratings (Spearman’s *ρ* = .40, p < .001).

**Conclusion**: Our rating scale is a reliable and applicable assessment tool. The rating scale focuses on the evaluation of general communication skills and can be applied in research as well as in evaluations, such as objective structured clinical examinations (OSCE).

**Abbreviations**: CST: Communication skills training; ICC: Intra-class correlation coefficient; OSCE: Objective structured clinical examination; SP: Standardized patients; SD: Standard deviation; M: Mean

## Introduction

A new awareness regarding the importance of good communication in the medical field has grown steadily over the last 20 years [1–4]. In response, recommendations and guidelines [5–8] as well as research on teaching communication skills, have become focus areas.

**Figure 1.**
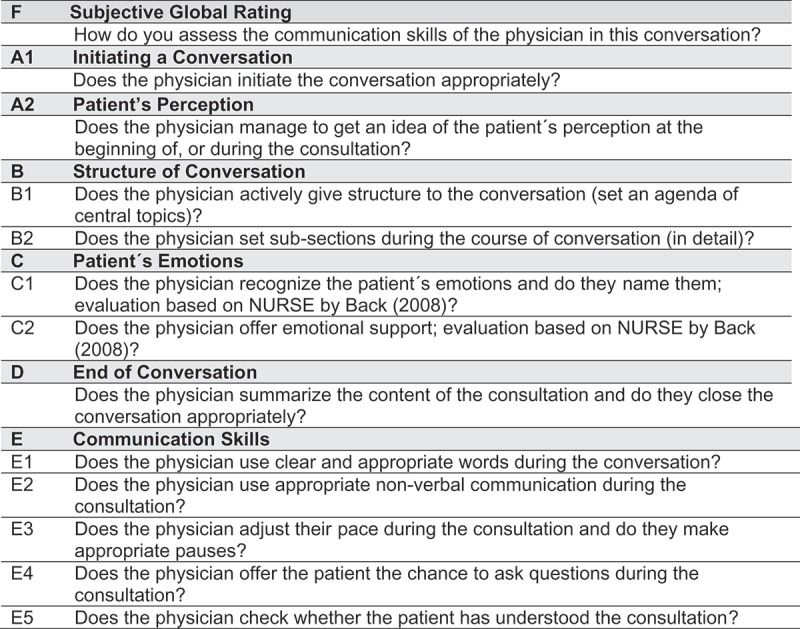
Coding system items of the ComOn check rating scale

Studies have described positive effects from patient-centered communication for both patients and clinicians. For patients, these positive effects include higher satisfaction with care, greater adherence to treatment, reduced anxiety, increased information, and improved understanding (e.g., [9,10]). For clinicians, it includes greater job satisfaction, better time management, and lower burnout levels [9]. In addition, an improvement in the physician-patient relationship and treatment as a whole, i.e., patient health outcomes, occurs [11,12]. In contrast more ‘physician-centered’ communication might lead to insufficient detection of psychological disturbances, patients’ dissatisfaction with care, and poorer compliance [13].

Many concepts for communication skills training (CST) for physicians (e.g., [14–19]), medical students (e.g., [20–23]), and nurses (e.g., [15,24–26]) have been presented and evaluated. Recent reviews [27,28] and a meta-analysis [29] report that CST shows effects with small to medium effect size in the short-term, while long term effects tend to be small. Whereas this indicates that communication skills, in principle, can be taught successfully, we see two questions in the research field that are not satisfactorily answered: (1) how to teach and transfer communication skills into practice, and (2) how to adequately assess these skills.

Efforts have been undertaken in order to enhance the transfer from CST into clinical practice including methods such as individual supervision both in person [18,19,24,30] and by the telephone [26], consolidation workshops [17], complementary sessions on stress management [16], and video-conferences [14]. Also, Curtis et al. [15] substituted a longer CST workshop with several shorter training-blocks to enhance the transfer into clinical practice.

The development of instruments for assessing physician-patient encounters has two main challenges. The first challenge is operationalization of good communication, which addresses the definition of observable criteria that allows an evaluation of good communication as objectively as possible. Secondly, the aim of evaluation focuses on settings, goals, and demands. Therefore, an assessment instrument must meet different requirements depending on the settings, for example for detailed interaction research or for an evaluation of CST. The assessment instrument must have various goals, for example the analysis of verbal or non-verbal communication or providing feedback for students. Finally, the instrument must have to meet different demands, for example either good psychometric properties, feasibility, or a combination and balance of the two. It will be difficult for one single instrument to meet all of these requirements.

As a result, many instruments have been developed. The *Roter Interaction Analysis System* (RIAS; [31,32]), the *Medical Interaction Process System* (MIPS, which was adapted from the RIAS for oncology settings; [33]), and the *CN-Logit* (later renamed *CANCODE*; [34,35]) are well-established instruments that show good validity and reliability and thus are often used in research. Similar systems are the *Cancer Research Campaign Workshop Evaluation Manual* (CRCWEM; [17,36]), the *LaComm* computer program [16], and the developed VR-CoDES, which especially focus on addressing emotional cues and concerns [37].

While these interaction analysis systems allow a very detailed data analysis, their application is very resource-intensive and time-consuming. Some focus on transcripts only and do not capture non-verbal behaviors, thereby missing an important part of communication [38]. Their emphasis lies on socioemotional behavior and less on specific communication skills to achieve a specific goal with the consultation [39] and are inadequate for evaluating changes in concrete communication skills. Therefore, there is a need for a more time-efficient and easily applicable instrument.

More recently developed instruments addressed these issues. For example the *SEGUE* Framework tool [10,40] is an acronym describing six steps: *S*et the stage, *E*licit information, *G*ive information, *U*nderstand the patients’ perception, and *E*nd the encounter. It is commonly used for internal medicine residents and is well-established in North America for teaching, assessing, and researching. The *CoMeD-OSCE* [41], which stands for Communication in Medical Education Düsseldorf – Objective Structured Clinical Examination, evaluates undergraduate medical students after they take part in the *CoMeD* training. The *Frankfurt Observer Communication Checklist* (FrOCK [42];) is also a very efficient rating tool for communication skills performance of medical students during an exam within a limited time. The *Gap-Kalamazoo Communication Skills Assessment Form* [43], an adaptation of the *Kalamazoo Consensus Statement Checklists* [3,43], specifically targets the assessment of communication skills of multidisciplinary clinicians. The *MAAS-Global Rating List* [44] with 47 items assessing communication skills and clinical examination skills as well as the Calgary Cambridge Guides [45] with 28 items are both rather extensive.

While these instruments and coding systems report solid reliability and validity, most of them are either limited in their application to certain doctor-patient conversations or for student examination and teaching/feedback purposes. Most do not have a satisfying balance between efficacy and informative value. Some of the instruments are only capable of analyzing transcripts from audiotaped consultations, thereby not taking into account the various non-verbal aspects of good communication. Therefore, there is a need for an instrument that assesses a broader spectrum of communication.

Additionally, several authors suggest that there is often a mismatch between stated behaviors and the inventories or procedures used to assess them [46,47].

These problems led CST researchers to focus on the development of a more specific rating scale, i.e., adapted to their teaching goals, that would still enable quantitative ratings. The coding system developed by Brown and Bylund [48] allows a quantitative analysis of the skills taught during training by analyzing video recordings whereas the system by Butow et al. [14] additionally enables a qualitative rating for some items. With this approach, students and raters get detailed information about their perfomance that can easily be used for CST purposes and exams. Our research group has also successfully used this approach in the past [49]. However, this developed rating scale was content-specific for oncology and in its original form could not be used for other consultation types. Therefore, we sought to overcome the shortcoming of existing instruments by developing a new rating scale that assesses a broad spectrum of communication skills and is efficient, straightforward, and for general use.

The study was approved by the Ethics Committee of the TUM School of Medicine, Germany (Project Number 5816/13). All students gave their informed consent to be videotaped during this encounter.

## Methods

### Aim of the study

The aim of the study is to present the development and evaluation process of a tool to assess communication skills of medical students. We wanted to integrate the issues of general application, efficiency, purpose, detailed evaluation, setting, good psychometric properties, and qualified raters. Later, we will describe psychometric properties.

#### General application

The purpose of the new rating scale is to have an assessment and evaluation tool for a wide range of settings and topics, suitable for medical students.

#### Efficiency

We wanted to develop a rating scale that allowed on-the-spot ratings during doctor-patient interactions in order to be used in teaching contexts to provide quick and efficient feedback. For this purpose, communication skills have to be assessed efficiently (within 5–10 minutes). Therefore, the rating scale needed to be manageable and clearly structured.

#### Detailed evaluation

At the same time we wanted to provide not only global assessments, but also specific information. We included into the coding system the main factors of good communication skills confirmed by the majority of other instruments. We scaled these on a five-point Likert scale to get a quantitative image of the diverse tasks. The ratings needed to be detailed enough to allow a meaningful evaluation of the outcome of a training course, i.e., the rating scale should not only code the frequency of a particular communication skill, but also a differentiation if a skill approved. We also aimed to provide change-sensitive measures.

#### Rated behavior

The instrument needed to be able to evaluate video recorded data and should permit the analysis of verbal, paraverbal (tone, pitch, and pacing of the voice), and non-verbal (gesture, mimic) elements of communication.

#### Specific setting

The rating scale should directly correspond to the specific skills taught in training courses [46,47]. On the other hand, the use of the application should not be limited to a specific physician-patient setting (e.g., oncology, exams) but should be applicable in a broad range of medical settings.

#### Psychometric properties

The instrument needed to conform to common standards of test quality criteria including objectivity, reliability, responsiveness/sensitivity to change, and validity. This requires the development of an informative instruction manual for the training of raters.

#### Qualified raters

Ratings should only be undertaken by trained experts because patients’ satisfaction ratings have repeatedly shown high ceiling effects [50,51], and patients’ or students’ ratings are considered to be highly subjective in nature [52].

### Design of the study: development of the ratings scale ComOn check

This study describes the process of the ratings scale development, finally named *ComOn check*. Furthermore, we evaluated the psychometric properties of the final version. The development of the rating scale included ten steps:

1. We first integrated relevant items from a previous rating scale, the *COM-ON-Checklist* [49]. This rating scale already covered the main factors relevant for general and content-specific communication skills and was created in the framework of two CST evaluation studies [18,19] in order to evaluate consultations of oncologists with their patients in two different scenarios: the shift from curative to palliative treatment, and disclosing information about clinical trials. The *COM-ON-Checklist* is based on the *SPIKES Model* [53] as this was the theoretical background of the teaching content. *SPIKES* structures and exemplifies good communication in six steps. It stands for S = Setting up the interview, P = assessing patient’s Perception, I = obtaining the patient’s Invitation, K = giving Knowledge and information to the patient, E = addressing the patient’s Emotions with Empathic responses, and S = Strategy and Summary. It was originally established for the physicians’ task of conveying bad news in oncology, but can be easily adapted for different contexts [18,19]. We calculated an item analysis and then excluded non-reliable items from the checklist. The rating scale was added with the skills presented in the coding system by Brown and Bylund [48]. We built up on these rating scales and continued with the development of our new rating scale, the ComOn check.
2. Next, we reviewed current literature and added important items in order to create a first version of our new checklist for general consultations. We integrated a method of structuring consultations presented by Langewitz et al. [54] using the ‘book metaphor.’ The authors elaborate on how to structure a consultation by naming the topic (name of the book), presenting the agenda (book chapters), explaining options (content of a chapter), and ending with a summary. For addressing emotions, we incorporated the *NURSE* model by Back et al. [55]. They operationalized empathy using the steps of *N*aming emotions, *U*nderstanding, *R*especting, *S*upporting, and *E*xploring. We focused on verbalizing emotions as well as showing respect and understanding for patients’ feelings.
3. We phrased items and scaled them according to a five-point Likert scale: 0 (poor) to 4 (excellent) points. In that way the checklist could assess individual improvements in communication skills for each physician focusing on observable behaviors. In the end, our checklist consists of a global rating and 12 specific items relating to the following aspects and subscales (see Figure 1):

**A1. Patient’s perception**

**A2. Patient’s perception**

**B. Structure of conversation**

(B1 Setting an agenda and B2 Structure in subchapters)

- **(C) Patient’s emotions**

(C1 Naming/understanding patient’s emotions, and C2 Respecting/supporting/exploring patient’s emotions)

**(D) End of conversation**
**(E) Communication skills** (E1 Use of clear language, E2 Adequate non-verbal communication, E3 Pauses, E4 Offer to ask questions, and E5 Check for understanding)
**(F) Global rating**

A first version of the items was presented in Niglio de Figueiredo et al. [56]. Although we decided to treat items A1 and A2 as different domains, we kept their names unchanged so that the development process would be more comprehensible.

Descriptions for ratings of 0 (poor), 2 (ok), and 4 (excellent) were defined as anchor points. Finally, we described in detail how to apply the checklist in a manual.

- In the next step, two psychologists who were the future raters reviewed the checklist. For the review process, we created a feedback sheet for the raters to formulate points of clarification for each item. These comments were discussed in a group and the amendments were integrated into the next version of the checklist.
- Next, we used videotaped consultations of first year medical students who had limited prior training in communication skills. Raters were instructed to watch this sample of videotaped consultations twice. The first time to assess the specific skills (A–E) and the second time to assess general communication skills (F) giving a comprehensive evaluation. Unclear items and their corresponding anchor points were critically discussed and revisions were made accordingly.
- Then, 20 videotaped consultations were rated using this second coding system. The two raters assessed ten videos together, followed by a discussion, and then ten videos separately. We analyzed inter-rater reliability and modified items with obvious inter-rater agreement. In addition, we changed the order the items should be rated: first, the raters gave a subjective global rating about the conversational competence of the physician, and second, they assessed the general and specific communication skills.
- Using this version the same 20 videotaped consultations were rated again. Experimentally they were watched three times instead of two to achieve more reliability. Inter-rater correlation and Cohen’s kappa were analyzed and the results were discussed in a group. However, watching three times did not give better results in rating consensus. Minimal modifications of the anchor points were made to create checklist version four.
- The focus was now on generating concurrence between the two raters. The discussion of differences between raters aimed at identifying and modifying items with a broad scope of interpretation to improve reliability across raters.
- Coding system version four was used to rate another 20 videotaped consultations, followed by a discussion and calculation of the inter-rater correlation. Lastly, modifications on the anchor points of the checklist were integrated to the final version five of the checklist.
- The last step was to define the final anchor points 0, 2, and 4 (see appendix). The final version of the coding system allowed the rating of 135 videotaped consultations, which was done individually by each rater. Divergence between ratings were discussed and settled by a consensus agreement. These values were used in the future analyses.

Final inter-rater agreement (as stated in the results section) was calculated from the ratings according to the final version of the rating scale.

The development of the ratings scale was in German and was translated for this publication.

### Setting of the study

One aim of the new rating scale *ComOn Check* was to apply this scale later to analyze 135 videotaped consultations, which were collected as part of a randomized controlled trial testing the effectiveness of a new teaching concept [57]. Students in the first clinical year at our medical school were told that there was to be a study to test a new educational concept, and that participation was voluntary, but they were blind to any details concerning its content. Students were assigned to take a history of a standardized patient that was unknown to them.

### Characteristics of participants

There were 69 students randomly assigned to one of two training groups and both groups were to evaluate an innovative teaching course compared to an existing course. Three participants withdrew because of illness, but the rest completed the study protocol as planned. Thus, we were able to analyze 135 videotaped consultations of 66 students (*M* = 21.9 years old, SD = 2.0; 75.9% female). Each student in each group had videotaped pre- and post- assessments of a communication task carried out with standardized patients (SP). Six SPs had been trained in advance to act out the patient’s role similarly but with flexibility to the students’ communication behavior. The students’ assignment was to have initial contact with the patient in the general practitioner role and to take his/her history. Time for consultations was limited to five minutes. At the end of the consultation, the SPs evaluated students’ global performances using a single item rating instrument in the form of a 10-cm visual analogue scale ranging from ‘0 – very bad performance’ to ‘10 – excellent performance.’ These ratings from the SPs were a criterion for the assessment of external validity. The two raters were experienced psychologists in primary patient care and trained in coping with errors of psychological ratings, for example the halo-effect, the primacy-recency effect, and the baseline error.

### Process, comparison, and statistical analyses

Although the consensus rating was applied in the final analyses, inter-rater reliability between the two raters was evaluated after rating all 135 videos. First, the percentage of absolute agreement between both raters was estimated. Second, a two-way mixed model was used to calculate intra-class correlation coefficients (ICCs) to assess consistency between raters. Cohen’s Kappa, a widely used measure of inter-rater agreement, does not provide information on the source of disagreement Therefore, we preferred to calculate the ICC because it also indicates how reliably raters agree while being less influenced by systematic rater-related error, e.g., one rater generally being ‘more strict’ in his evaluations. For all analyses described below, the rating values derived from consensus agreements were used. Descriptive statistics for all items and subscales as well as a total sum score (including all items except for the raters’ global assessment) are presented as mean, range, and standard deviation. As a measure of item selectivity, item-total parametric correlations were calculated for all items A1 – E5 and the global rating F with their total sum score. Furthermore, internal consistency of the subscales (B – *Structure of conversation*, C – *Patient’s emotions*, and E – *Communication skills*) was evaluated using Cronbach’s *α*. A Spearman’s rank correlation matrix is provided to show associations between the subscales as well as items A1, A2, and D. Spearman’s correlation coefficient between SP-rated student performance and overall expert ratings was calculated as an indicator of external validity. Correlation coefficients were considered small if |r| ≥ .10; medium if |r| ≥ .30; and large if |r| ≥ .50. All statistical analyses were done using the Statistical Package for Social Sciences (SPSS, version 22).

## Results

### Evaluation of inter-rater reliability

As shown in Table 1, we found an absolute agreement of 31–77% depending on the different items. The average ICCs indicated moderate (items *B1, B2,* and *C1*) to high (items F, *A1, C2, D*, and *E1-E5*) inter-rater reliabilities with the exception of item A2 (*Patient’s perception*), which despite a medium-high rater absolute agreement, was characterized by a low ICC.

**Table 1.** Inter-rater reliability of rating scale items

	Inter-rater reliability	
Item	Agreement (%)	ICC
F – Global rating	45	.744
A1 – Initiating a conversation	77	.758
A2 – Patient’s perception	46	.405
B1 – Setting an agenda	37	.569
B2 – Structure in subchapters	31	.525
C1 – Naming/understanding patient’s emotions	33	.641
C2 – Respecting/supporting/exploring patient’s emotions	48	.713
D – End of conversation	53	.701
E1 – Use of clear words	52	.821
E2 – Adequate non-verbal communication	63	.842
E3 – Pauses	52	.793
E4 – Offer to ask questions	71	.829
E5 – Check for understanding	49	.710

### Description of rating scale items and sub-scores

Table 2 summarizes descriptive statistics of consensus ratings for all rating scale items as well as a total sum score and subscales. One item (D) could only be rated in 130 of the 135 cases because the end of the conversation was not recorded in five videotapes.

**Table 2.** Coding system, item-total correlations, and internal consistency of sub-scores

		Coding system		Item-total correlations	Internal consistency
Item/scale	N	Range	Mean (SD)	Pearson correlation coefficient	Cronbach’s *α*
F – Global rating	135	0–4	2.24 (1.19)	–	–
A1 – Initiating a conversation	135	0–4	2.11 (1.04)	0.15	–
A2 – Patient’s perception	135	0–4	2.91 (.87)	0.31*	–
B1 – Setting an agenda	135	0–4	2.53 (1.27)	0.62*	–
B2 – Structure in subchapters	135	0–4	2.21 (1.32)	0.78*	–
C1 – Naming/understanding patient’s emotions	135	0–4	2.24 (1.27)	0.68*	–
C2 – Respecting/supporting/exploring patient’s emotions	135	0–4	2.67 (1.45)	0.70*	–
D – End of conversation	130	0–4	2.00 (.98)	0.44*	–
E1 – Use of clear words	135	0–4	2.59 (1.31)	0.70*	–
E2 – Adequate non-verbal communication	135	0–4	3.10 (.90)	0.62*	–
E3 – Pauses	135	0–4	2.59 (1.31)	0.71*	–
E4 – Offer to ask questions	135	0–4	1.93 (.92)	0.53*	–
E5 – Check for understanding	135	0–4	2.45 (1.22)	0.64*	–
B – Structure of conversation	135	0–8	4.75 (2.44)	–	0.87
C – Patient’s emotions	135	0–8	4.91 (2.51)	–	0.82
E – Communication skills	135	3–20	12.66 (4.12)	–	0.77
					
Sum score (A1 – E5)	135	9–44	29.36 (8.17)	[1]	0.83

*p < 0.01.

#### Item level

The whole Likert scale, from 0 to 4 points, was fully utilized in all the items. Item-total correlations showed significant associations between all items A1-E5 and their sum score with the exception of item A1. Pearson correlation coefficients ranged from 0.31 (A2 – *Patient’s perception*) to 0.78 (B2 – *Structure in subchapters*). The accordance for item F (*Global rating for total assessment*) with the sum score was nearly perfect, with a correlation coefficient of 0.91.

#### Sub-score level

With regard to the three subscales, an exhaustive range of possible scores was achieved, with the exception that no student was rated to have very low overall communication skills (subscale E, range 3–20). Cronbach’s *α* of the domains B, C, and E as well as the overall sum score (A1-E5) indicated acceptable to good internal consistencies with values greater than 0.77.

Table 3 represents the correlations of the subscales B, C, and E as well as the discrete items A1, A2, and D. Whereas the items A1 and A2 did not correlate with any other item or subscale, associations between the domains B, C, E, and item D were small to large. The strongest association could be found between subscales C and E (*ρ* = 0.59, p < 0.001), indicating that their proportion of shared variance is about 35%.

**Table 3.** Correlations between the subscales B, C, and E and specific items A1, A2, and D.

		B	C	D	E	F
**A1 –** Initiating a conversation	Spearman’s *ρ*	−0.01	0.05	<0.01	<0.01	0.01
	*p value*	*0.94*	*0.54*	*0.97*	*0.98*	*0.87*
**A2 –** Patient’s perception	Spearman’s *ρ*		0.13	0.15	0.04	0.22
	*p value*		*0.131*	*0.085*	*0.619*	*0.011*
**B –** Structure of conversation	Spearman’s *ρ*			0.44**	0.30**	0.52**
	*p value*			*<0.001*	*<0.001*	*<0.001*
**C –** Patient’s emotions	Spearman’s *ρ*				0.35**	0.59**
	*p value*				*<0.001*	*<0.001*
**D –** End of conversation	Spearman’s *ρ*					0.28*
	*p value*					*0.001*
**E –** Communication skills						
**F –** Global rating						

*p < 0.01; **p < 0.001.

### External validity criterion

We used SP global ratings of student performance as criteria for external validity. N = 135 ratings averaged a score of *M *= 7.52 (*SD* = 1.806), covering a range of 1.1 – 10.0.

There was a moderate association between the two variables of interest, i.e., SP ratings and item F (Global rating for total assessment), (*ρ* = 0.40, *p* < 0.001).

## Discussion

### Summary of results

We developed a rating scale to assess aspects of physician-patient communication that are relevant in different settings of health-care encounters. We focused only on general communication skills and developed a rating scale independent from specific context. The items of the rating scale represent key verbal and non-verbal communication skills discussed in the current literature. The rating scale enables a quantitative and qualitative assessment approach and it is time-efficient. Improvements in communication skills can be assessed on a five-point Likert scale, which also enables assessment of small changes. Additionally, it is widely applicable, time-efficient, and can be used to evaluate students’ performance in OSCEs. Statistical quality criteria are met because our rating scale provides objectivity by providing precise behavioral anchor point descriptions for each item. Reliability is good, as indicated by our high internal consistency and acceptable inter-rater reliability. Internal validity is warranted by deriving our items from current literature. External validity is ensured by a statistically significant correlation with our external criteria, i.e., evaluation from our SP. Although the rating scale assesses partially independent skills, the combination of these skills seems to improve the communication competence, as shown by a high correlation between the sum score and global assessment of the encounter.

In comparison to other behavior-oriented task-focused instruments, our ratings scale offers a differentiated approach to assessing communication skills without losing attention to detail. The five-point Likert scale can assess a more sensitive change than a three-point scale as it is applied in the Calgary Cambridge Guide [45]. The 13 developed items comprise key points of patient-centered communication, while being focused and time-efficient. This is harder to fulfill with a 47-item rating scale such as the MAAS-Global rating list [44] or in a detailed interaction analysis (e.g. the RIAS [31,32];). Moreover, our rating scale was conceived in a way that should allow assessments in general settings as well as in other, more specific, but not strictly content-specific, settings.

### Strengths and limitations

Any psychometric instrument is challenged to be objective, valid, and reliable and at the same time applicable and time-efficient. Our priority was to have a simple rating scale for both quick and precise evaluation with acceptable statistical properties. While statistical quality criteria are met in most respects, inter-rater reliability as indicated by the ICCs was only in the acceptable range. The problem of low intra-class correlation has been discussed with regard to the Calgary Cambridge Guide [45], which had ICCs between 0.05 and 0.57 for their rating scale. They concluded that their results are acceptable against the background of the complexity of professional communication. We found ICCs between 0.41 and 0.84 in our study, which can therefore be deemed as sufficiently good. We used as outside criterion a single item assessed by our SP. A more elaborate assessment could strengthen the external validity of the rating scale. With regard to proper doctor-patient encounters, Kurtz et al. [58] emphasized the need of combining content (i.e., medical subject matter expertise) and process variables (i.e., communication skills). While we agree with that statement, the presented rating scale focuses on communication skills, only. However, we hypothesize that our rating scale is applicable for a variety of settings. We are testing this feature of our rating scale in a parallel project, for which a study protocol has already been published [56].

## Conclusion

Our newly developed rating scale, *ComOn check*, is an applicable and convenient instrument. Although communication characteristics are diverse and multi-dimensional, the developed rating scale provides a good evaluation of communication performance. It can be implemented not only in research, but also in evaluating students’ performance on an OSCE. During the development of the instrument we worked with professional raters who needed training to ensure high quality ratings. In the future the applicability of *ComOn check* in teaching projects and OSCEs should be assessed. The applicability during real life encounters of physicians with their patients (study protocol already published [56];) and its change-sensitivity for measuring communication performance over time requires evaluation [59]. Future research should also prove its cross-cultural applicability. The checklist manual including behavior-based anchor points for all items is available from the corresponding author upon request. Readers are invited to freely utilize the checklist in both teaching and research settings.

## Data Availability

The data are available from the author or are attached in the appendix.
